# Src-family kinase-Cbl axis negatively regulates NLRP3 inflammasome activation

**DOI:** 10.1038/s41419-018-1163-z

**Published:** 2018-10-31

**Authors:** I-Che Chung, Sheng-Ning Yuan, Chun-Nan OuYang, Hsin-Chung Lin, Kuo-Yang Huang, Yu-Jen Chen, An-Ko Chung, Ching-Liang Chu, David M. Ojcius, Yu-Sun Chang, Lih-Chyang Chen

**Affiliations:** 1 Molecular Medicine Research Center, Chang Gung University, Taoyuan, 333 Taiwan; 2 Graduate Institute of Medical Sciences, National Defense Medical Center, Taipei, 114 Taiwan; 3 Division of Clinical Pathology, Department of Pathology, Tri-Service General Hospital, Taipei, 114 Taiwan; 4 Graduate Institute of Pathology and Parasitology, National Defense Medical Center, Taipei, 114 Taiwan; 5 Department of Medical Research, Mackay Memorial Hospital, New Taipei City, 251 Taiwan; 6 Department of Radiation Oncology, Mackay Memorial Hospital, New Taipei City, 251 Taiwan; 7 Graduate Institute of Biomedical Sciences, College of Medicine, Chang Gung University, Taoyuan, 333 Taiwan; 8 Graduate Institute of Immunology, College of Medicine, National Taiwan University, Taipei, 100 Taiwan; 9 Department of Biomedical Sciences, University of the Pacific Arthur A. Dugoni School of Dentistry, San Francisco, CA 94103 USA; 10 Center for Molecular and Clinical Immunology, Chang Gung University, Taoyuan, 333 Taiwan; 11 Chang Gung Immunology Consortium, Chang Gung Memorial Hospital, Linkou, 333 Taiwan; 12 Department of Otolaryngology-Head & Neck Surgery, Chang Gung Memorial Hospital, Linkou, 333 Taiwan; 13 Department of Medicine, Mackay Medical College, New Taipei City, 252 Taiwan

## Abstract

Activation of the NLRP3 inflammasome is crucial for immune defense, but improper and excessive activation causes inflammatory diseases. We previously reported that Pyk2 is essential for NLRP3 inflammasome activation. Here we show that the Src-family kinases (SFKs)-Cbl axis plays a pivotal role in suppressing NLRP3 inflammasome activation in response to stimulation by nigericin or ATP, as assessed using gene knockout and gene knockdown cells, dominant active/negative mutants, and pharmacological inhibition. We reveal that the phosphorylation of Cbl is regulated by SFKs, and that phosphorylation of Cbl at Tyr371 suppresses NLRP3 inflammasome activation. Mechanistically, Cbl decreases the level of phosphorylated Pyk2 (p-Pyk2) through ubiquitination-mediated proteasomal degradation and reduces mitochondrial ROS (mtROS) production by contributing to the maintenance of mitochondrial size. The lower levels of p-Pyk2 and mtROS dampen NLRP3 inflammasome activation. In vivo, inhibition of Cbl with an analgesic drug, hydrocotarnine, increases inflammasome-mediated IL-18 secretion in the colon, and protects mice from dextran sulphate sodium-induced colitis. Together, our novel findings provide new insights into the role of the SFK-Cbl axis in suppressing NLRP3 inflammasome activation and identify a novel clinical utility of hydrocortanine for disease treatment.

## Introduction

Inflammasomes are cytoplasmic multiprotein complexes that are important for innate immunity. They comprise various cytosolic pattern recognition receptors, such as nod-like receptors (NLRPs) and AIM2, along with the adaptor protein, ASC, and pro-caspase-1^1^. Inflammasome assembly triggers an ASC-mediated oligomerization that results in formation of large “speck” structures^2^. The ASC specks are required for activation of caspase-1, which mediates the maturation of interleukin (IL)-1β and IL-18 and ultimately pyroptotic cell death. The NLRP3 inflammasome can be stimulated by pathogen-associated molecular patterns and damage-associated molecular patterns (e.g., nigericin^3^ and ATP^4–6^). Activation of the NLRP3 inflammasome has also been shown to be critically involved in maintaining epithelial integrity in the colon and attenuating dextran sulfate sodium (DSS)-induced colitis in mice^7,8^. However, excessive activation of the NLRP3 inflammasome is responsible for progression of several inflammation-associated diseases, including cryopyrin-associated periodic syndrome^9^, septic shock^10^, rheumatoid arthritis^11^, Alzheimer’s disease^12^, and type 2 diabetes^13^. At present, it is unclear how the inflammasome is regulated to prevent excessive inflammation under normal conditions.

The NLRP3 inflammasome can respond to various types of stimuli, which can activate the inflammasome via kinase activity and mitochondrial reactive oxygen species (mtROS) production^6,14^. We previously showed that the protein tyrosine kinase, Pyk2, critically mediates NLRP3 inflammasome activation by directly phosphorylating ASC at Tyr146^15,16^. However, it is not known how Pyk2 itself is regulated. One potential upstream regulator is Cbl, which has been reported to inhibit mtROS production^17^. Many lines of evidence indicate that the NLRP3 inflammasome can be activated by mtROS^6^, but there is still a missing link between the regulatory kinases and mtROS production.

Cbl is encoded by a proto-oncogene and is a RING finger E3 ubiquitin ligase^18^. Cbl negatively regulates many phosphotyrosine signaling pathways via its N-terminal tyrosine kinase-binding domain; this allows it to interact with phosphotyrosine-containing proteins, which it ubiquitinates and thereby targets for proteasomal or lysosomal degradation. Cbl also participates in the maintenance of mitochondrial function and size. Cbl-knockout (KO) mice display an increase in whole-body energy expenditure along with mitochondrial hypertrophy in skeletal muscle^19^. Although tyrosine phosphorylation and mtROS are known to regulate NLRP3 inflammasome activation, the potential role of Cbl remains unclear. The E3 ligase activity of Cbl is regulated through phosphorylation by Src-family kinases (SFKs)^20^, and SFKs have been implicated in NLRP3 inflammasome activation^21,22^, thus suggesting a link.

In this study, we show that SFK-Cbl axis plays a role in suppressing the NLRP3 inflammasome. In addition, the analgesic drug, hydrocotarnine, can block Cbl activity, therefore enhancing NLRP3 inflammasome activation. In the DSS-induced animal model of colitis, treatment with hydrocotarnine increased IL-18 secretion and protected mice from the disease. Together, our results provide important new insights into the biological processes responsible for preventing excessive inflammation.

## Materials and methods

### Reagents, antibodies, and plasmids

PMA (phorbol 12-myristate 13-acetate), ATP, nigericin, CCCP, PP2, dAdT, and MG132 were purchased from Sigma. MitoSox, H_2_-DCFDA, TMRE, MitoTracker Green FM, and Hoechst were purchased from Life Technologies. MitoTEMPO was purchased from Enzo Life Sciences. Hydrocotarnine was purchased from Enamine. Anti-Pyk2, anti-AIM2, and anti-p-Pyk2 were purchased from Cell Signaling. Anti-Cbl, anti-ASC, anti-caspase-1, anti-IL-1β, anti-phosphotyrosine, and anti-GAPDH were purchased from Santa Cruz. Anti-Ly6G, anti-CD45, and anti-CD11b were purchased from BD Bioscience. Anti-NLRP3 and anti-F4/80 were purchased from BioLegend and eBioscience, respectively. Plasmids encoding mutants CBL (Y371D) and CBL (Y371F) were generated by ligating amplified DNA fragments into the *Nhe*I/*Pme*I-treated pLKO_AS2.neo vector (RNAi Core, Taiwan). The mutant CBL was constructed using a QuikChange II Site-Directed Mutagenesis kit (Agilent Technologies), and the following primers (forward and reverse, respectively): for Cbl Y371D, 5′-CAGGAACAATATGAATTAGACTGTGAGATGGGCTCCAC-3′ and 5′-GTGGAGCCCATCTCACAGTCTAATTCATATTGTTCCTG-3′; and for Cbl Y371F, 5′-CAGGAACAATATGAATTATTCTGTGAGATGGGCTCCAC-3′ and 5′-GTGGAGCCCATCTCACAGAATAATTCATATTGTTCCTG-3′.

### Flow cytometric isolation of ASC-mCherry speck-forming cells

The ASC-mCherry speck-forming cells were isolated by flow cytometry as described by Sester et al.^23^. Briefly, nigericin-treated THP-1-ASC-mCherry cells were gated, analyzed for their inflammasome activation state by pulse width to pulse area profile (W:A) analysis, and sorted for the presence/absence of ASC-mCherry specks.

### Animal experiments

Mouse experiments were performed under the ethical approval by the Institutional Animal Care and User Committee of Chang Gung University, and the methods were carried out in accordance with the approved guidelines. C57BL/6 mice were obtained from National Laboratory Animal Center, Taiwan. Hck/Fgr/Lyn^−/−^ triple KO (TKO) mice were kindly provided by Dr. C. L. Chu (Graduate Institute of Immunology, College of Medicine, National Taiwan University, Taipei, Taiwan)^24^. All mice were maintained under specific pathogen-free conditions and used at 6–9 weeks of age. For lipopolysaccharide (LPS)-induced endotoxin shock, mice were intraperitoneally treated with 1 or 10 mg/kg of LPS as indicated. At 24 h after LPS treatment, peripheral blood cells were collected. Neutrophils (CD45^+^/Ly6G^+^) and macrophages (CD45^+^/F4/80^+^) were subjected to antibody staining and analyzed with a FACSCalibur (Becton Dickinson). To generate the DSS-induced colitis model, 2.5% DSS (molecular weight, 36 000–50 000; MP Biomedicals) was given to mice via the drinking water. To evaluate the protective effect of Cbl on DSS-induced colitis, mice were dosed with 10 mg/kg of hydrocotarnine by intraperitoneal injection daily from day 0 to day 9. Treatment with 2.5% DSS began on day 1 and ended on day 7. Body weight was assessed daily. Mice were sacrificed at day 10. Colon length measurement and colon organ culture were performed. For histopathological analysis, colons were fixed, embedded, sectioned, and stained with hematoxylin and eosin. The histopathology was scored with respect to inflammation, epithelial defects, and crypt atrophy, as previously described^25^.

### Cell culture

Bone marrow cells were collected from the tibias and femurs of wild-type (WT) and TKO C57BL/6 mice by flushing with cold phosphate-buffered saline (PBS) using a 25-G needle, and mouse bone marrow-derived macrophages (BMDMs) were generated and cultured in Dulbecco’s modified Eagle medium supplemented with 10% fetal calf serum and 10 ng/ml macrophage colony-stimulating factor (PeproTech) for 8 days. The THP-1 (human leukemia monocytic) cell line was purchased from the Biosource Collection and Research Center (Taiwan) and maintained in RPMI as described previously^26^. The ASC-mCherry-expressing THP-1 cells were kindly provided by Dr. M. Z. Lai (Institute of Molecular Biology, Academia Sinica, Taipei, Taiwan)^27^. For macrophage differentiation, THP-1 cells were stimulated with 200 nM PMA for 16 h. For inflammasome activation, the cells were treated with 10 μM nigericin for 0.5 or 1 h as indicated, with 5 mM ATP for 4 h, with 200 μg/ml monosodium urate (MSU) for 4 h, or with 2 μg/ml poly(dA:dT) for 4 h. For inhibition of signaling or caspase activity, the cells were pretreated with PP2 (10 μM), Z-VAD-FMK (20 μM), MitoTEMPO (1 μM), MG132 (3 μM), or hydrocotarnine (10 μM) for 1 h, and then subjected to inflammasome stimulation. The Cbl-KO HEK293T cell line was generated using the CRISPR/Cas9 system with dual small guide RNAs targeting Cbl (CTCGGCTCGACTGCGAGCGA and GTCCACCGTCCCCGGCGGGT). For the reconstitution of NLRP3 inflammasomes, WT and Cbl-KO HEK293T cells were co-transfected with pLKO_AS2-Flag-NLRP3, pLKO_AS2-ASC-Flag, pLKO.1-IL-1β, or pCMV6-Entry-Casp-1 using Lipofectamine 2000 (Invitrogen) as previously described^16^. The cells were then incubated for 48 h and analyzed as described for the HEK293T cells. For Cbl reconstitution, Cbl-KO HEK293T cells were transduced with lentiviral vectors encoding mutant Cbl (Y371D or Y371F) and selected with puromycin.

### RNA interference

The double-stranded RNA duplexes were purchased from Dharmacon and transfected into cells using Lipofectamine 2000, as previously described^28^. For efficient knockdown, the cells were incubated for 2 days. The reagent used to target Cbl included three 19-bp RNA duplexes: 5′-GACAAUCCCUCACAAUAAA-3′, 5′-UAGCCCACCUUAUAUCUUA-3′, and 5′-GGAGACACAUUUCGGAUUA-3′.

### Immunoblot analysis

Cells were lysed in RIPA buffer (50 mM Tris-Cl, pH 7.5, 150 mM NaCl, 10 mM MgCl_2_, 1 mM EDTA, and 1% Igepal CA-630) with a protease inhibitor cocktail (4.76 μg/ml leupeptin, 3.25 μg/ml aprotinin, 0.69 μg/ml pepstatin, and 1 mM phenylmethylsulfonyl fluoride) on ice for 30 min. For assessment of IL-1β secretion and caspase-1 activation, culture supernatants were collected, mixed with a 1/10 volume of 100% (wt/vol) trichloroacetic acid, and incubated for 10 min at 4 °C. The precipitated protein samples were resolved by SDS-polyacrylamide gel electrophoresis (SDS-PAGE) and transferred to nitrocellulose membranes (Amersham). The membranes were incubated with the indicated primary antibodies, and then with an horseradish peroxidase (HRP)-conjugated secondary antibody. The immunoreactive bands were detected using TOOLS Extreme ECL-HRP Substrate (BIOTOOLS CO., LTD. Taiwan). Immunoblot images were quantified with the ImageJ software.

### Immunoprecipitation

Cells were lysed in RIPA buffer with a protease inhibitor cocktail, and cell extracts (1 mg) were immunoprecipitated with anti-Pyk2 antibodies (1 μg) for 24 h. The corresponding rabbit IgGs (Millipore) were used as the control antibodies. The bound samples were precipitated with PureProteome Protein G Magnetic Beads (Millipore) for 1 h at 4 °C, and the immunoprecipitated products were collected for immunoblot analysis.

### ASC oligomerization assay

THP-1 cells were lysed in buffer A (20 mM HEPES-KOH, pH 7.5, 10 mM KCl, 1.5 mM MgCl_2_, 1 mM EDTA, 1 mM EGTA, and 320 mM sucrose) supplemented with a protease inhibitor cocktail. Nuclei and unlysed cells were removed by centrifugation through 5 μm Ultrafree-CL centrifugal filters (Millipore). The filtrate was diluted with an equal volume of CHAPS buffer (20 mM HEPES-KOH, pH 7.5, 5 mM MgCl_2_, 0.5 mM EGTA, and 0.1% CHAPS) supplemented with a protease inhibitor cocktail, and centrifuged to obtain the insoluble pelleted fraction. The pellets were resuspended in CHAPS buffer and then subjected to crosslinking using 2 mM disuccinimidyl suberate for 30 min. The protein samples were resolved by 12% SDS-PAGE, and the level of ASC was analyzed. ASC speck formation was analyzed in mouse BMDMs and ASC-mCherry-expressing THP-1 cells. ASC speck images were acquired under fluorescence microscopy (Olympus). For quantification, the ASC specks were counted with an IN Cell Analyzer (GE Healthcare) and normalized with respect to the number of nuclei, which were stained with 4′,6-diamidino-2-phenylindole.

### Enzyme-linked immunosorbent assay

Cell culture supernatants and mouse sera were assayed for human IL-1β (eBioscience), human IL-18 (R&D Systems), and mouse IL-1β (eBioscience), respectively. Colon organ culture supernatants were assayed for mouse IL-18 (R&D Systems).

### Mitochondrial and cellular ROS measurements

Mitochondrial and cellular ROS were measured using MitoSox Red and H_2_-DCFDA (Life Technologies), respectively. The cells were incubated with 5 μM MitoSox Red or 10 μM H_2_-DCFDA at 37 °C for 20 min, and then analyzed by flow cytometry.

### Confocal microscopy

Cells were treated with MitoTracker Green FM (200 nM) and Hoechst (5 μg/ml) for 30 min. To measure the cellular distribution of mitochondria, images were obtained with a confocal laser scanning microscope (Carl Zeiss, LSM780) and processed with the ZEN microscopic software (Carl Zeiss).

### Transmission electron microscopy

The cells were fixed in 3% glutaraldehyde and 2% paraformaldehyde in 0.1 M cacodylate buffer (pH 7.4) for 2 h at 4 °C. The cells were then postfixed in 1% osmium tetroxide (pH 7.4), dehydrated in a graded ethanol series, and embedded in EPON-812 (Nacalai Tesque, Japan). Thin sections (80 nm) were cut, stained with uranyl acetate and lead citrate, and examined on an H-7500 EM transmission electron microscope (Hitachi). Micrographs (×9000−×12 000) were analyzed with the ImageJ software (version 1.51s), which was used to manually trace the mitochondrial outer membrane area and cytoplasm area for calculation of the number, average size (μm^2^), and volume density (% of cytoplasm) of mitochondria. Eleven and 16 randomly selected cells were examined from the WT and Cbl-KO groups, respectively.

### Statistical analysis

All statistical analyses were performed using the SPSS 13.0 statistical software package (SPSS, Inc.). Data from in vitro experiments and tumor growth in the mouse model were analyzed with the Student’s *t* test. Differences were considered significant at *P* < 0.05.

## Results

### Cbl inhibits NLRP3 inflammasome activation

**Fig. 1:**
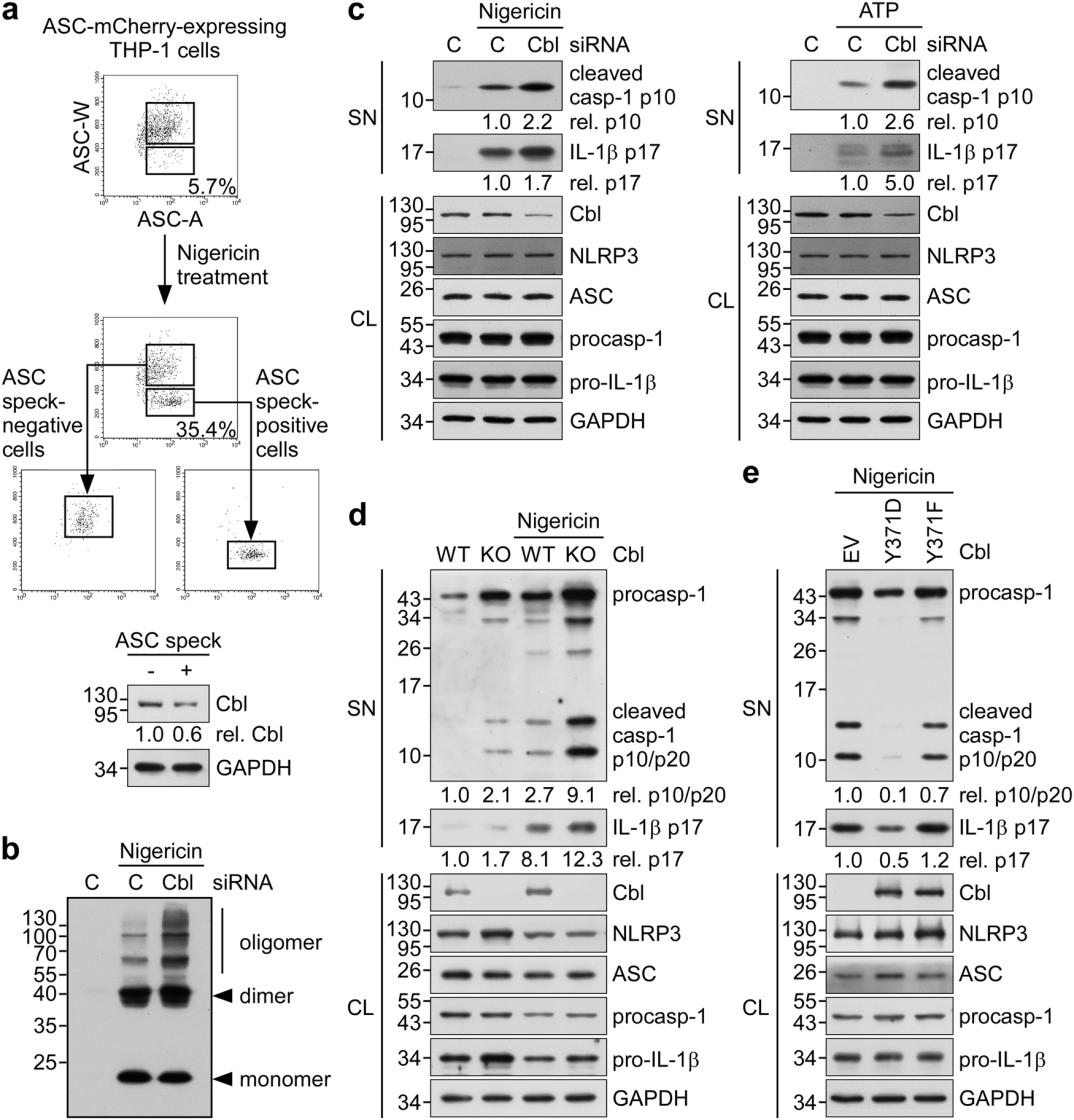
Phosphorylation of Cbl at Y371 is required to suppress the NLRP3 inflammasome. **a** ASC-mCherry-expressing THP-1-derived macrophages were treated with nigericin for 1 h, ASC speck-containing cells were isolated by flow cytometry (low and high ASC-W:ASC-A profiles were taken as indicating ASC speck-positive and -negative cells, respectively), and immunoblotting was performed using anti-Cbl and anti-GAPDH antibodies. **b** Analysis of ASC oligomerization in THP-1-derived macrophages that were treated with Cbl siRNA or negative control siRNA (C) and stimulated with nigericin for 1 h. **c** THP-1-derived macrophages were transfected with Cbl siRNA or negative control siRNA (C) and treated with nigericin for 1 h or ATP for 4 h, and culture supernatants (SN) and cell lysates (CL) were immunoblotted with antibodies that recognize Cbl, GAPDH, and NLRP3 inflammasome molecules. **d** NLRP3 inflammasome activation was analyzed in reconstituted WT and Cbl-KO HEK293T cells that were co-transfected with vectors encoding Flag-NLRP3, ASC-Flag, IL-1β, or caspase-1 for 48 h, treated with nigericin for 5 h, separated into SN and CL fractions, and immunoblotted with antibodies that recognize Cbl, GAPDH, and NLRP3 inflammasome molecules. **e** Cbl-KO HEK293T cells stably expressing empty vector (EV) or mutant CBL (Y371D or Y371F) were treated with nigericin for 5 h, and SN and CL were immunoblotted with antibodies that recognize Cbl, GAPDH, and NLRP3 inflammasome molecules. The western blot is a representative of three independent experiments. procasp-1, p45 precursor of caspase-1; cleaved Casp-1, p20 and p10 active caspase-1 subunits; IL-1β p17, secreted mature IL-1β; and pro-IL-1β, p31 precursor of IL-1β

We previously showed that p-Pyk2 enables NLRP3 inflammasome activation through the direct phosphorylation of ASC at Tyr146^16^. However, much less is known about the degradation of p-Pyk2, which leads to suppression of NLRP3 inflammasome activity. Cbl is a phosphotyrosine-interacting protein that targets proteins with its ubiquitin ligase activity to route them for proteasomal degradation^29^. Here we examined the role of Cbl in suppressing NLRP3 inflammasome activation. During formation of the NLRP3 inflammasome complex, ASC molecules form a speck and oligomerize to recruit pro-caspase-1 prior to the activation of caspase-1. To quantify inflammasome formation in ASC speck-positive and -negative cells after stimulation, we carried out time-of-flight inflammasome evaluation analysis^23^. In ASC-mCherry-expressing THP-1 cells treated with the NLRP3 agonist, nigericin, the percentage of ASC speck-containing cells was increased to 35.4% compared with the 5.7% seen in untreated cells (Fig. 1a, top). Notably, the level of Cbl protein was 0.6-fold in ASC speck-containing cells relative to ASC speck-negative cells (Fig. 1a, bottom). This suggested that there was a higher Cbl protein level in cells that failed to efficiently form ASC specks. We further examined whether Cbl depletion affects ASC oligomerization, and found that nigericin induced more ASC oligomers in THP-1 cells treated with a Cbl-specific small interfering RNA (siRNA) compared to those treated with control siRNA (Fig. 1b). To evaluate whether Cbl is involved in regulating the NLRP3 inflammasome, we analyzed changes in caspase-1 activation and IL-1β maturation in cells treated with nigericin or ATP (both agonists of the NLRP3 inflammasome). The levels of mature IL-1β p17 and active caspase-1 (as assessed by the level of caspase-1 p10) were increased in both Cbl-knockdown THP-1 cells (Fig. 1c) and NLRP3 inflammasome-reconstituted Cbl-KO HEK293T cells achieved by CRISPR/Cas9 technology (Fig. 1d). However, Cbl knockdown in THP-1 cells did not change the amounts of poly(dAdT)-induced mature IL-1β, cleaved caspase-1, or ASC oligomers (Supplementary Figure S1). These results suggest that the ligase activity of Cbl specifically suppresses the NLRP3 inflammasome, but not the AIM2 inflammasome.

### Phosphorylation of Cbl at Y371 is required to inhibit NLRP3 inflammasome activation

The ligase activity of Cbl is regulated by a conformational change that is triggered by the phosphorylation of Cbl at Tyr371^29,30^. Thus, we hypothesized that the phosphorylation could govern the ability of Cbl to suppress NLRP3 inflammasome activation. We evaluated the production of mature IL-1β and cleaved, activated caspase-1 in NLRP3 inflammasome-reconstituted Cbl-KO HEK293T cells stably expressing constructs encoding constitutively active Cbl (Cbl Y371D) or dominant-negative Cbl (Cbl Y371F) in the presence and absence of nigericin stimulation^31^. As shown in Fig. 1e, the ability of nigericin stimulation to induce mature IL-1β and cleaved caspase-1 was dramatically repressed in cells expressing constitutively active Cbl (Y371D), but not in the cells expressing dominant-negative Cbl (Y371F). These results strongly support the notion that Tyr371 phosphorylation and ligase activity of Cbl contribute to regulation of the NLRP3 inflammasome.

### Cbl inhibits Pyk2 signaling via ubiquitination-mediated proteasomal degradation

**Fig. 2:**
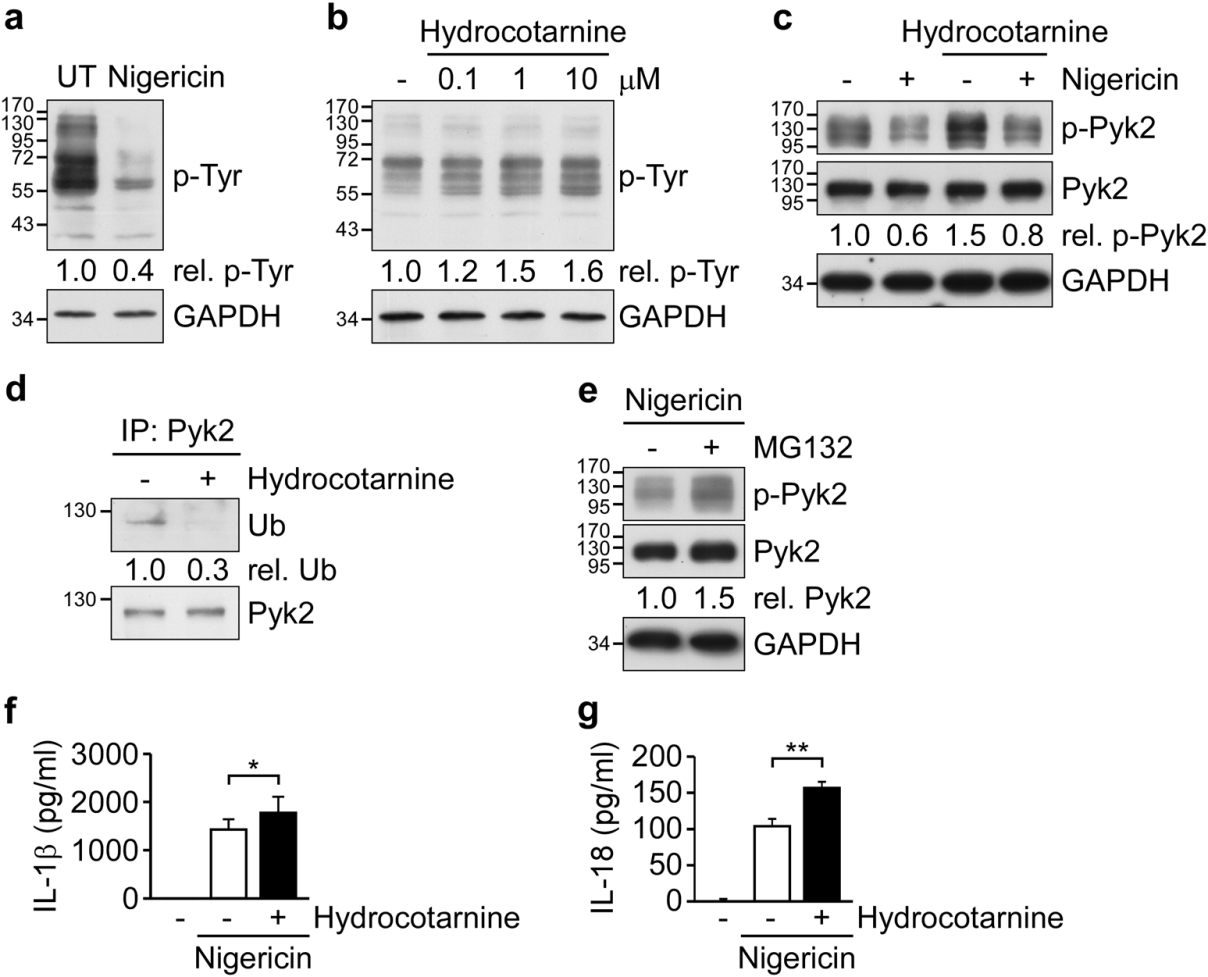
Cbl inhibits Pyk2 signaling via ubiquitination-mediated proteasomal degradation. **a** THP-1-derived macrophages were left untreated (UT) or treated with nigericin for 1 h, and tyrosine-phosphorylated proteins (p-Tyr) were detected by immunoblotting with anti-p-Tyr antibodies. **b** THP-1-derived macrophages were treated with 0.1, 1, or 10 μM hydrocotarnine for 1 h and immunoblotted with anti-p-Tyr and anti-GAPDH. **c** THP-1-derived macrophages were pretreated with 10 μM hydrocotarnine for 1 h, treated with nigericin for 1 h, and subjected to immunoblotting with anti-p-Pyk2, anti-Pyk2, and anti-GAPDH antibodies. **d** THP-1-derived macrophages were pretreated with 10 μM hydrocotarnine for 1 h, and cell lysates were subjected to immunoprecipitation (IP) using anti-Pyk2 followed by immunoblotting with anti-ubiquitin (Ub) and anti-Pyk2 antibodies. **e** THP-1-derived macrophages were pretreated with MG132 for 1 h, treated with nigericin for 1 h, and subjected to immunoblotting with anti-p-Pyk2, anti-Pyk2, and anti-GAPDH antibodies. The western blot is representative of three independent experiments. **f**, **g** THP-1-derived macrophages were pretreated with 10 μM hydrocotarnine for 1 h and then treated with nigericin for 1 h, and supernatants were subjected to IL-1β (**f**) and IL-18 (**g**) ELISA. *P < 0.05; **P < 0.01. All results are presented as the mean ± SD of three independent experiments, and were analyzed with the Student’s *t* test

Since Pyk2 is critical for NLRP3 inflammasome activation^16^ and has been detected in the Cbl-interactome^32,33^, we speculated that Cbl may suppress the NLRP3 inflammasome through negative regulation of Pyk2. To address this possibility, we first examined the global pattern of tyrosine-phosphorylated proteins during NLRP3 inflammasome activation. As shown in Fig. 2a, stimulation with nigericin significantly decreased the global level of tyrosine-phosphorylated proteins by 0.4-fold. Treatment with ATP showed similar results (Supplementary Figure S2). In addition, treatment with the Cbl-specific inhibitor, hydrocotarnine (CRIN-2, patent number: WO2011160016A2), dose-dependently increased the global level of tyrosine-phosphorylated proteins (Fig. 2b) and induced p-Pyk2 (Fig. 2c) in THP-1 cells. Notably, hydrocotarnine treatment attenuated the reduction of p-Pyk2 seen following NLRP3 activation (Fig. 2c) and decreased the ubiquitination of Pyk2 (Fig. 2d). Thus, Cbl contributes to negative regulation of p-Pyk2 through ubiquitin-mediated proteasomal degradation. This possibility was supported by our observation that p-Pyk2 is increased in THP-1 cells treated with the proteasome inhibitor, MG132 (Fig. 2e). Moreover, the secretion of IL-1β and IL-18 was significantly elevated in hydrocotarnine-pretreated THP-1 cells compared to control cells (Fig. 2f,g and Supplementary Figure S3). Taken together, our results suggest that Cbl likely suppresses the NLRP3 inflammasome due to negative regulation of Pyk2.

### Cbl maintains mitochondrial size and reduces ROS production

**Fig. 3:**
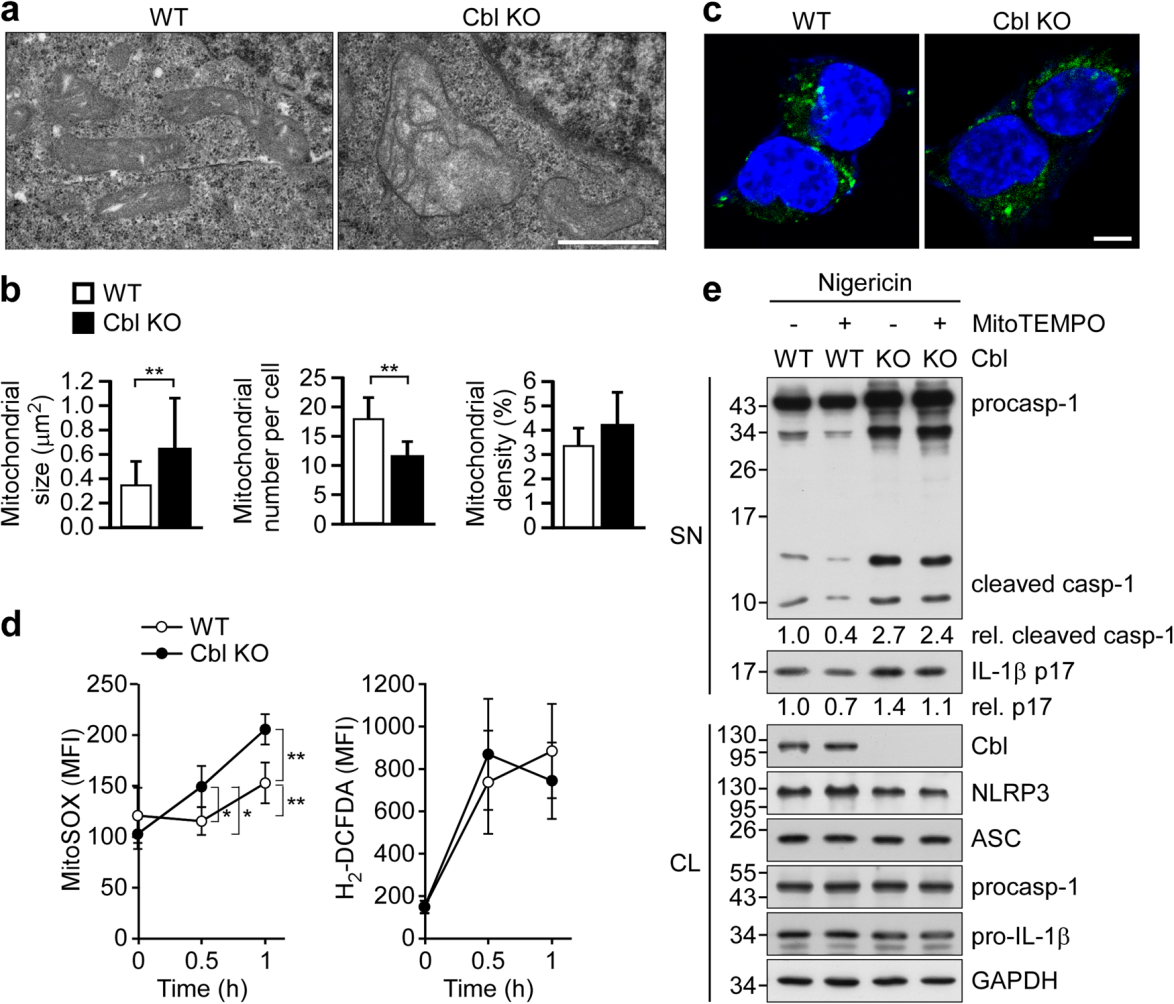
Cbl maintains mitochondrial size and reduces mtROS production. **a** Representative electron micrographs of WT and Cbl-KO HEK293T cells treated with nigericin for 5 h. Magnification, ×75 000; scale bar, 100 nm. **b** Quantification of the average size (μm^2^), number and volume density (% of cytoplasm) of mitochondria from WT and Cbl-KO HEK293T cells treated with nigericin for 5 h. Eleven and 16 cells were randomly examined from the WT and Cbl-KO cell groups, respectively. **c** Representative confocal images of the cellular distribution of mitochondria in WT and Cbl-KO HEK293T cells treated with nigericin for 5 h. Mitochondria are shown in green, while nuclei are blue. Scale bar, 5 μm. **d** Productions of mitochondria and cellular ROS by WT and Cbl-KO HEK293T cells treated with nigericin for 0.5 or 1 h were measured using MitoSOX and H_2_-DCFDA, respectively. *P < 0.05; **P < 0.01. All results are presented as the mean ± SD of three independent experiments, and were analyzed with the Student’s *t* test. **e** WT or Cbl-KO HEK293T cells were pretreated with MitoTEMPO for 1 h and treated with nigericin for 5 h, and culture supernatants (SN) and cell lysates (CL) were subjected to immunoblotting with antibodies that recognize Cbl, GAPDH, and NLRP3 inflammasome molecules. The western blot is a representative of three independent experiments. procasp-1, p45 precursor of caspase-1; cleaved Casp-1, p20 and p10 active caspase-1 subunits; IL-1β p17, secreted mature IL-1β; and pro-IL-1β, p31 precursor of IL-1β

In addition to Pyk2 signaling, NLRP3 inflammasome activation is regulated by mitochondrial dysfunction and downstream mtROS production^34^. In Cbl-deficient mice, the mitochondrial size is reportedly enlarged in muscle tissues^19^. To determine if the same might be true in our in vitro system, we used electron microscopy to analyze the structure of mitochondria in Cbl-KO HEK293T cells. We found that the average size of mitochondria was increased (Fig. 3a, b, left), but the average number of mitochondria per cell was decreased in Cbl-KO HEK293T cells compared with WT HEK293T cells (Fig. 3b, middle). There was no difference between Cbl-KO and WT cells with respect to mitochondrial density (Fig. 3b, right) or the cellular distribution of mitochondria (Fig. 3c). Next, we examined whether Cbl might also affect mitochondrial health. In response to the NLRP3 activator, nigericin, both Cbl-KO and WT cells exhibited increased production of mtROS and cellular ROS (Fig. 3d). Notably, a significantly higher induction of mtROS production was observed in Cbl-KO cells compared with WT cells (Fig. 3d, left). Consistent with this increase in mtROS production, higher levels of mature IL-1β and cleaved caspase-1 were observed in Cbl-KO cells compared with WT cells in response to NLRP3 inflammasome activation, and these effects were partially inhibited by pretreatment with the mtROS scavenger, MitoTEMPO (Fig. 3e). These data suggested that Cbl is required to maintain mitochondrial size and function, which critically contribute to NLRP3 inflammasome activation by modulating mtROS production.

### SFK-mediated Cbl phosphorylation inhibits NLRP3 inflammasome activation

**Fig. 4:**
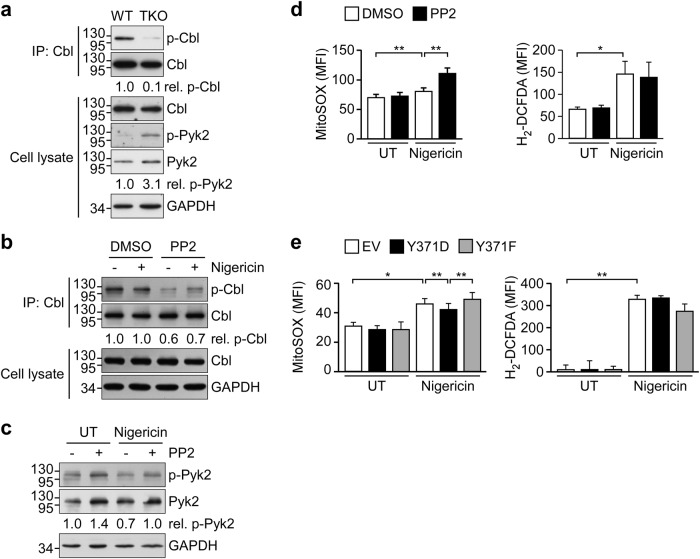
Cbl activity is regulated by SFKs. **a** Cell lysates obtained from bone marrow-derived macrophages (BMDMs) of WT C57BL/6 and TKO mice were subjected to IP using anti-Cbl and immunoblotted with anti-phosphotyrosine, anti-Cbl, anti-p-Pyk2, anti-Pyk2, and anti-GAPDH antibodies. **b**, **c** THP-1-derived macrophages were pretreated with PP2 or DMSO for 1 h and treated with nigericin for 1 h, and cell lysates were subjected to IP and immunoblotting with the indicated antibodies. The western blot is representative of three independent experiments. **d** THP-1-derived macrophages were pretreated with PP2 or DMSO for 1 h and treated with nigericin for 15 min, and productions of mitochondria and cellular ROS were measured using MitoSOX and H_2_-DCFDA, respectively. **e** Cbl-KO HEK293T cells stably expressing empty vector (EV) or mutant CBL (Y371D or Y371F) were treated with nigericin for 1 h and subjected to measurement of mitochondria and cellular ROS. *P < 0.05; **P < 0.01. All results are presented as the mean ± SD of three independent experiments, and were analyzed with the Student’s *t* test

As ubiquitination of Cbl requires the prior phosphorylation of Tyr371 by SFKs^20^ and our present results indicate that Cbl critically suppresses the NLRP3 inflammasome, we examined whether regulator SFKs, as upstream regulators of Cbl, might suppress the NLPR3 inflammasome. As shown in Fig. 4a, the levels of Cbl phosphorylation were decreased by 0.1-fold in BMDMs from TKO mice deficient in the three SFKs, Hck, Fgr, and Lyn. In contrast, the level of Pyk2 phosphorylation was markedly increased by 3.1-fold in BMDMs from TKO mice. Treatment of THP-1 cells with the SFK-specific inhibitor, PP2, reduced Cbl phosphorylation in the absence and presence of nigericin by 0.6- and 0.7-fold, respectively, compared to the dimethyl sulfoxide control (Fig. 4b). Moreover, PP2 treatment induced Pyk2 phosphorylation from 1.0- to 1.4-fold and from 0.7- to 1.0-fold in the untreated and nigericin-treated groups, respectively (Fig. 4c). We also examined whether mtROS production might be regulated by SFKs in response to NLRP3 inflammasome activation. As shown in Fig. 4d, the level of mtROS induced by nigericin was higher in PP2-pretreated THP-1 cells, but there was no change in cellular ROS production. As our data indicated that reduced mtROS production and phosphorylation of Cbl at Y371 are required for the Cbl-mediated negative regulation of NLRP3 inflammasome activation, we further investigated whether Tyr371 phosphorylation of Cbl is required for mtROS production. As shown in Fig. 4e, the nigericin-triggered induction of mtROS was repressed in HEK293T cells expressing constitutively active Cbl (Y371D) but not dominant-negative Cbl (Y371F), whereas these cells did not differ in their production of cellular ROS. These results reveal that Pyk2 activity and mtROS production are all regulated by SFKs and the phosphorylation of Cbl at Y371 may participate in Cbl-mediated mtROS production.

**Fig. 5:**
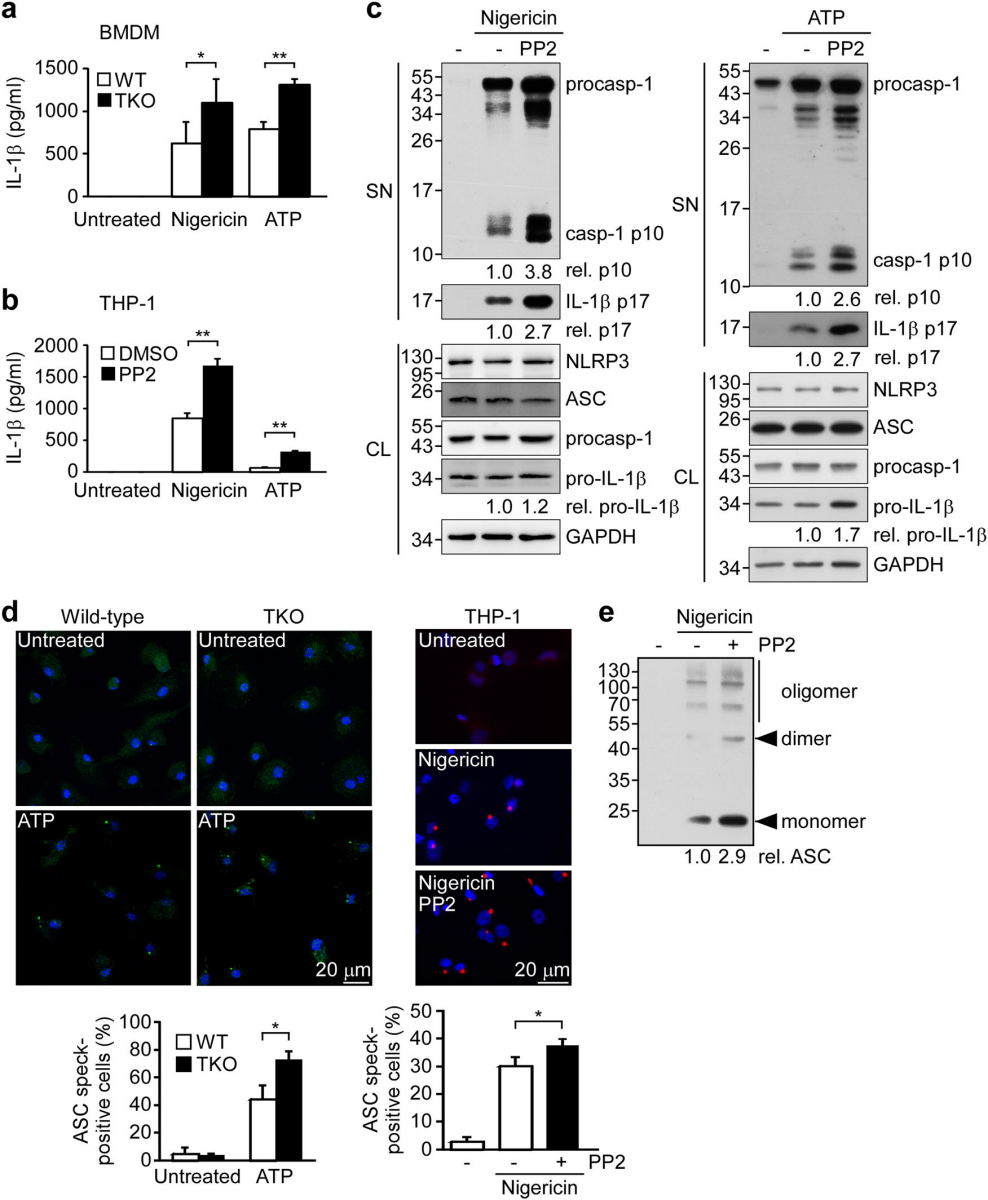
SFKs inhibit the NLRP3 inflammasome. BMDMs from WT C57BL/6 and TKO mice (**a**) or PP2-preteated THP-1 derived macrophages (**b**) were treated with nigericin for 1 h or ATP for 4 h and supernatants were subjected to IL-1β ELISA. **c** THP-1-derived macrophages were pretreated with or without PP2 for 1 h and treated with nigericin for 1 h or ATP for 4 h, and SN or CL were subjected to immunoblotting with antibodies that recognize GAPDH and NLRP3 inflammasome molecules. **d** Left panel: BMDMs from WT and TKO mice were treated with ATP for 4 h and ASC speck formation was visualized by immunostaining with an anti-ASC antibody. Right panel: PMA-differentiated ASC-mCherry-expressing THP-1 cells were pretreated with PP2, and then treated with nigericin for 1 h. ASC is shown in green (left panel) or red (right panel), while nuclei are shown in blue. Scale bars, 20 μm. The bottom panels present the percentages of cells with ASC specks, as determined by an IN Cell Analyzer. **e** Analysis of ASC oligomerization in THP-1-derived macrophages pretreated with PP2 and stimulated with nigericin for 1 h. procasp-1, p45 precursor of caspase-1; cleaved Casp-1, p20 and p10 active caspase-1 subunits; IL-1β p17, secreted mature IL-1β; and pro-IL-1β, p31 precursor of IL-1β. *P < 0.05; **P < 0.01. These data are representative of three independent experiments

We further tested the effect of SFK deficiency and/or inhibition on activation of the NLRP3 inflammasome. We found that the induction of IL-1β secretion by nigericin or ATP was increased in BMDMs from TKO mice (Fig. 5a) and PP2-pretreated THP-1 cells (Fig. 5b), suggesting that SFKs may suppress nigericin- or ATP-induced NLRP3 inflammasome activation. Consistent with this notion, the levels of mature IL-1β p17 and active caspase-1 p10 induced by nigericin or ATP were increased in PP2-pretreated THP-1 cells (Fig. 5c). After ATP stimulation, the percentage of ASC speck-containing cells was increased from 44.4% in BMDMs from WT mice to 72.6% in those from TKO mice (Fig. 5d, left). In ASC-mCherry-expressing THP-1 cells, PP2 treatment increased the proportion of nigericin-induced ASC speck-containing cells from 30.1 to 37.7% (Fig. 5d, right). The nigericin-induced oligomerization of ASC in THP-1 cells was also further increased by PP2 treatment (Fig. 5e). The above-described SFK-Cbl-mediated inflammasome regulation was not detected during poly(dAdT)-induced AIM2 inflammasome activation in BMDMs or THP-1 cells, nor in PP2-treated THP-1 cells or BMDMs from WT or TKO mice (Supplementary Figure S4). These findings indicate that the SFK-mediated phosphorylation of Cbl is involved in suppressing the NLRP3 inflammasome but not the AIM2 inflammasome.

To determine the physiological significance of SFKs in NLRP3 inflammasome suppression in vivo, we tested the effect of SFK deficiency in the mouse model of LPS-induced septic shock. LPS challenge yielded a much higher mortality rate in TKO mice compared to WT mice (Supplementary Figure S5a). The percentage of neutrophils but not macrophages was higher in peripheral blood from TKO mice compared to WT mice (Supplementary Figure S5b). Importantly, serum IL-1β production was significantly elevated in TKO mice compared to WT mice after the peritoneal injection of LPS (Supplementary Figure S5c). These results indicated that SFKs protect mice from endotoxic shock by suppressing the LPS-induced activation of the NLRP3 inflammasome in vivo.

### Inhibition of Cbl attenuates acute ulcerative colitis

**Fig. 6:**
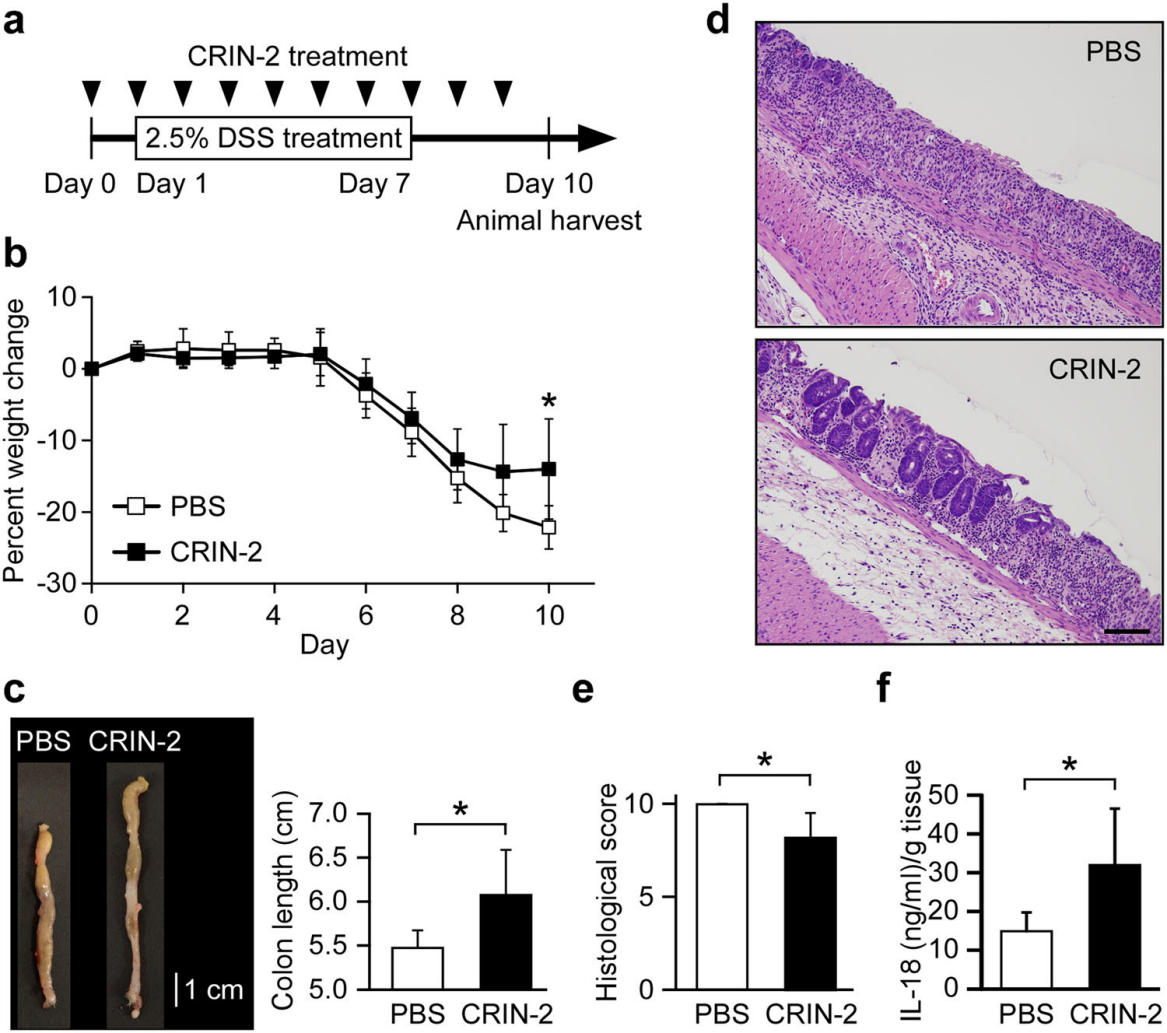
Cbl inhibitor treatment attenuates acute ulcerative colitis in a mouse model. **a** Schematic presentation of the DSS-induced mouse model of colitis. Mice were injected intraperitoneally with hydrocotarnine for 10 consecutive days (day 0 to day 9) and exposed to DSS or PBS (control) via their drinking water for 7 consecutive days (day 1 to day 7) to induce colitis. All mice were sacrificed on day 10; *n* = 5 mice/group. **b** Percent weight change was monitored daily. **c** Colon length. **d** Representative photomicrographs of H&E-stained colon sections; scale bars, 100 μm. **e** Histological scores were examined in hydrocotarnine-treated and PBS control mice on day 10. **f** ELISA of IL-18 from colon organ cultures obtained from hydrocotarnine-treated and PBS control mice on day 10. *P < 0.05

Activation of the NLRP3 inflammasome has been shown to critically maintain epithelial integrity in the colon and attenuate DSS-induced colitis in mice^7,8^. Therefore, we assessed whether decreasing negative regulation of the NLRP3 inflammasome activation could attenuate colitis in an animal model by treating DSS-exposed mice with the Cbl inhibitor, hydrocotarnine (Fig. 6a). Indeed, hydrocotarnine significantly attenuated the weight loss of mice with DSS-induced colitis compared to PBS-treated control mice (Fig. 6b).

To further assess the disease status, we examined the colon length, which can be used as an indicator of disease severity. Indeed, the colons of hydrocotarnine-treated mice appeared to be significantly longer than those of PBS-treated control mice (~6.1 vs. ~5.5 cm) (Fig. 6c). The histopathology score, which was determined by combining the individual scores of inflammation, epithelial defects, and crypt atrophy^25^, indicated that hydrocotarnine-treated mice presented with significantly lower scores than PBS-treated control mice (Fig. 6d, e). Since IL-18 production downstream of the NLRP3 inflammasome is critically involved in protection against colitis and colorectal tumorigenesis^35,36^, we further determine IL-18 production in colon. The level of IL-18 was also significantly enhanced in colon organ cultures from hydrocotarnine-treated mice compared with control mice (Fig. 6f), suggesting that NLRP3 inflammasome activation was enhanced in hydrocotarnine-treated mice. This result was consistent with the apparent ability of Cbl to inhibit NLRP3 inflammasome-mediated processing of IL-18.

## Discussion

**Fig. 7:**
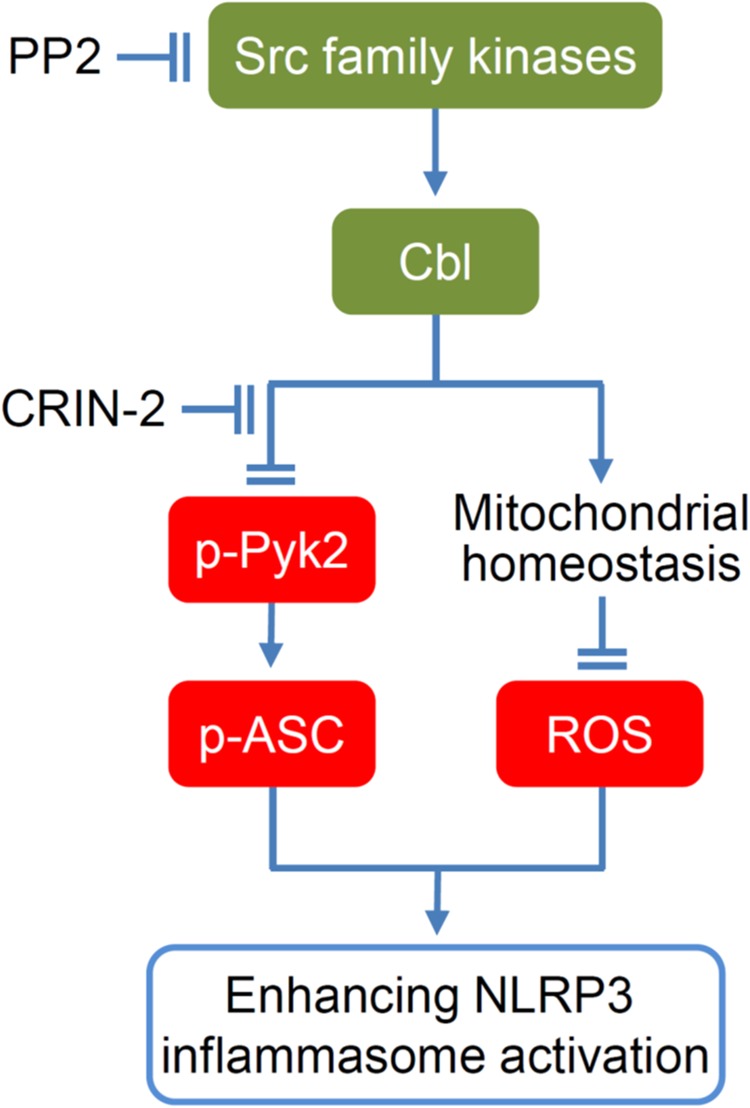
Model for SFK-Cbl axis-dependent negative regulation of the NLRP3 inflammasome. Based on our results, we propose that phosphorylation of Cbl is regulated by Src-family kinases, and that phosphorylation of Cbl at Tyr371 contributes to suppression of the NLRP3 inflammasome. The suppressive function of Cbl is dependent on its ability to downregulate p-Pyk2 via ubiquitination and proteasomal degradation, which reduces the Pyk2-mediated phosphorylation of ASC at Tyr146. Meanwhile, Cbl also reduces mtROS by maintaining homeostasis (appropriate size) of mitochondria. Both Tyr146-phosphorylated ASC and mtROS are negatively regulated by Cbl and are essential for NLRP3 inflammasome activation. Finally, the ability of Cbl to suppress NLRP3 inflammasome activation can be abrogated by the Cbl inhibitor, hydrocotarnine

Activation of the NLRP3 inflammasome is critical for immune defense, but improper and excessive activation can lead to inflammatory disease. Much remains to be learned about mechanisms that restrain inflammasome activation under normal conditions. Based on our present results, we propose a model for regulation of the NLRP3 inflammation by the SFK-Cbl axis (Fig. 7). Importantly, inhibition of Cbl with the pharmacological inhibitor, hydrocotarnine, enhances NLRP3 inflammasome activation and protects mice from DSS-induced colitis. Together, these novel results demonstrate a new mechanistic role for the SFK-Cbl axis in negatively regulating the NLRP3 inflammasome and reveal the availability of a novel treatment for colitis.

Recently, studies using pharmacological inhibitors have suggested that SFKs promote NLRP3-dependent IL-1β secretion in response to MSU^37^, malarial hemozoin^21^, influenza A virus^38^, and trichothecene mycotoxins^22^. Based on secretome analysis of MSU-stimulated macrophages, Nyman et al. reported that Src/Pyk2/PI3 kinase activity is required for the secretion of IL-1β and IL-18^37^. However, these studies did not provide direct evidence for the ability of SFKs to mediate inflammasome activation and caspase-1 cleavage. Here we confirm that SFKs promote MUS-induced IL-1β secretion in our system (Supplementary Figure S6), but our gene KO and pharmacological inhibition studies show that SFKs can also exert an opposing function by suppressing ATP- or nigericin-stimulated NLRP3 inflammasome activation. We found that SFK deficiency (in TKO mice) enhances the induction of IL-1β secretion and ASC speck formation in BMDMs and increases the LPS-induced mortality rate in vivo. The PP2-mediated inhibition of SFKs significantly increases caspase-1 cleavage, IL-1β maturation, ASC speck formation, and ASC oligomerization in THP-1-derived macrophages. Consistent with our findings, a previous study showed that deletion of the SFK, Lyn, can aggravate LPS-induced lung inflammation^39^. Importantly, we found that downregulation of SFK activity by gene KO or PP2 treatment was associated with a decrease in phosphorylated Cbl (p-Cbl) but increased levels of p-Pyk2 and mtROS, which are important mediators for NLRP3 inflammasome activation. Thus, SFKs may exert opposing functions in response to different stimuli for NLRP3 inflammasome activation. ATP activates the NLRP3 inflammasome through its receptor P2X7R, and both ATP- and nigericin-induced NLRP3 inflammasome activation requires potassium efflux^40^. As indicated in Fig. 7, the Src/Cbl/Pyk2 axis is required for regulation of the NLRP3 inflammasome in response to ATP and nigericin. On the other hand, MSU, which enters cells through phagocytosis, activates NLRP3 through Src/Pyk2/PI3 kinase^37^. We speculate that this discrepancy may result from the different downstream signal pathways that are involved in inflammasome activation in response to different types of stimuli.

Phosphorylation of Pyk2 is important for signal transduction^41,42^. However, the mechanism responsible for suppressing Pyk2 signaling remained unclear. Here we show that p-Pyk2 is downregulated by Cbl through ubiquitination and proteasome-dependent degradation. A recent study reported that the Cbl family member, Cbl-b, is involved in the mono-ubiquitination-mediated trypsin-induced degradation of Pyk2^43^. Here we show that the loss of the Cbl upstream activators, SFKs, is associated with a high level of p-Pyk2 and a correspondingly low level of p-Cbl, indicating that the SFK-Cbl axis is a negative regulator of Pyk2 signaling. In line with our previous report that Pyk2 is critical for NLRP3 inflammasome activation^16^, we found that gene KO or pharmacological inhibition of SFKs or Cbl both increases the level of p-Pyk2 and enhances NLRP3 inflammasome activation. Consistent with the ability of Cbl to suppress the NLRP3 inflammasome, depletion of Cbl-b reportedly increased inflammasome activation in response to infection by *Candida albicans*^44^. Together, these findings indicate that the SFK-Cbl axis suppresses the NLRP3 inflammasome through downregulation of Pyk2 signaling.

Enlarged mitochondria were previously observed in muscle cells from Cbl-deficient mice^19^. In agreement with the previous finding, we found that Cbl deficiency causes the size of mitochondria in HEK293T cells to increase. The mitochondrion is a dynamic organelle whose size is regulated by mitochondrial fusion/fission; these processes are critical for the elimination of damaged mitochondria under environmental stress^45^. Dysfunction of mitochondrial fusion/fission increases the presence of damaged mitochondria, which then become a major source of ROS. Several lines of evidence support the idea that mtROS is a direct activator of the NLRP3 inflammasome^6,46^. Consistent with this notion, we observed that nigericin stimulation increases mtROS production and NLRP3 inflammasome activation in Cbl-KO HEK293T cells, which harbor enlarged mitochondria. This observation agrees with a previous study showing that Cbl knockdown increases ROS production in T-cell lymphoma cells^17^.

Mitochondrial fission is mediated by Dyn2^47^, and Dyn2 depletion leads to enlargement of mitochondrial size^48^. Dyn2 also contributes to the Cbl-regulated degradation of the epidermal growth factor receptor (EGFR)^49^, and a Cbl-regulated interaction between Dyn2 and Cbl-interacting protein of 85K (CIN85) is important for EGFR endocytosis^50^. It has been speculated that Cbl may regulate mitochondrial size through the Dyn2-CIN85 complex. Here we provide new insights into the role of Cbl-regulated mitochondrial size and mtROS production in inhibition of the NLRP3 inflammasome.

Activation of the NLRP3 inflammasome is critical for the ability of an organism to fight antimicrobial infection^51–53^ and protect against some inflammation-related diseases, such as carcinogenesis during DSS-induced colitis^7,8,36^. The NLRP3 inflammasome is also involved in the ability of chemotherapeutics to act against tumors^28,54^. Here we show for the first time that the SFK-Cbl axis is involved in negatively regulating the NLRP3 inflammasome. Importantly, we show that inhibiting Cbl with the small molecule, hydrocotarnine, can increase the expression of p-Pyk2, which is essential for NLRP3 inflammasome activation by directly phosphorylating ASC^16^. Consistently, hydrocotarnine can enhance IL-18 secretion in response to NLRP3 inflammasome activation in vitro and enhance IL-18 secretion in vivo in the colon of mice with DSS-induced colitis. It is worth noting that hydrocotarnine can significantly attenuate weight loss, extent of colon shortening, and the histopathology score reflecting the protective effect of hydrocotarnine in DSS-induced colitis. Hydrocotarnine has been used clinically to enhance analgesic effects of oxycodone for the relief of pain and dyspnea in the patient with terminally ill cancer^55,56^, although the mechanism of action is mostly unknown^57^. Our data therefore suggest that the manipulation of Cbl with hydrocotarnine to enhance NLRP3 inflammasome activation could be relevant for the treatment of infectious disease, colitis, and cancer.

In summary, we herein propose a model for the previously unrecognized SFK-Cbl axis-dependent suppression of NLRP3 inflammasome activation. We show that SFK-Cbl activity increases the threshold for NLRP3 inflammasome activation by maintaining Pyk2 signaling and mtROS at appropriately low levels. Our findings suggest that Cbl should be considered as a new therapeutic target of hydrocotarnine in the treatment of inflammation-related diseases related to NLRP3 inflammasome activation.

## Electronic supplementary material

Supplemental Material

## References

[1] Broz P, Dixit VM (2016). Inflammasomes: mechanism of assembly, regulation and signalling. Nat. Rev. Immunol..

[2] Lu A (2014). Unified polymerization mechanism for the assembly of ASC-dependent inflammasomes. Cell.

[3] Munoz-Planillo R (2013). K(+) efflux is the common trigger of NLRP3 inflammasome activation by bacterial toxins and particulate matter. Immunity.

[4] Martinon F, Petrilli V, Mayor A, Tardivel A, Tschopp J (2006). Gout-associated uric acid crystals activate the NALP3 inflammasome. Nature.

[5] Gombault A, Baron L, Couillin I (2012). ATP release and purinergic signaling in NLRP3 inflammasome activation. Front. Immunol..

[6] Abais JM, Xia M, Zhang Y, Boini KM, Li PL (2015). Redox regulation of NLRP3 inflammasomes: ROS as trigger or effector?. Antioxid. Redox Signal..

[7] Allen IC (2010). The NLRP3 inflammasome functions as a negative regulator of tumorigenesis during colitis-associated cancer. J. Exp. Med..

[8] Zaki MH (2010). The NLRP3 inflammasome protects against loss of epithelial integrity and mortality during experimental colitis. Immunity.

[9] Cordero Mario D., Alcocer-Gómez Elísabet, Ryffel Bernhard (2018). Gain of function mutation and inflammasome driven diseases in human and mouse models. Journal of Autoimmunity.

[10] Lee S (2017). NLRP3 inflammasome deficiency protects against microbial sepsis via increased lipoxin B4 synthesis. Am. J. Respir. Crit. Care Med..

[11] Mathews RJ (2014). Evidence of NLRP3-inflammasome activation in rheumatoid arthritis (RA); genetic variants within the NLRP3-inflammasome complex in relation to susceptibility to RA and response to anti-TNF treatment. Ann. Rheum. Dis..

[12] Heneka MT (2013). NLRP3 is activated in Alzheimer’s disease and contributes to pathology in APP/PS1 mice. Nature.

[13] Lee HM (2013). Upregulated NLRP3 inflammasome activation in patients with type 2 diabetes. Diabetes.

[14] Neumann K, Ruland J (2013). Kinases conquer the inflammasomes. Nat. Immunol..

[15] Lin YC (2015). Syk is involved in NLRP3 inflammasome-mediated caspase-1 activation through adaptor ASC phosphorylation and enhanced oligomerization. J. Leukoc. Biol..

[16] Chung IC (2016). Pyk2 activates the NLRP3 inflammasome by directly phosphorylating ASC and contributes to inflammasome-dependent peritonitis. Sci. Rep..

[17] Wu J, Salva KA, Wood GS (2015). c-CBL E3 ubiquitin ligase is overexpressed in cutaneous T-cell lymphoma: its inhibition promotes activation-induced cell death. J. Invest. Dermatol..

[18] Mohapatra B (2013). Protein tyrosine kinase regulation by ubiquitination: critical roles of Cbl-family ubiquitin ligases. Biochim. Biophys. Acta.

[19] Molero JC (2004). c-Cbl-deficient mice have reduced adiposity, higher energy expenditure, and improved peripheral insulin action. J. Clin. Invest..

[20] Yokouchi M (2001). Src-catalyzed phosphorylation of c-Cbl leads to the interdependent ubiquitination of both proteins. J. Biol. Chem..

[21] Shio MT (2009). Malarial hemozoin activates the NLRP3 inflammasome through Lyn and Syk kinases. PLoS Pathog..

[22] Kankkunen P (2014). Trichothecene mycotoxins activate NLRP3 inflammasome through a P2X7 receptor and Src tyrosine kinase dependent pathway. Hum. Immunol..

[23] Sester DP (2015). A novel flow cytometric method to assess inflammasome formation. J. Immunol..

[24] Meng F, Lowell CA (1998). A beta 1 integrin signaling pathway involving Src-family kinases, Cbl and PI-3 kinase is required for macrophage spreading and migration. EMBO J..

[25] Meira LB (2008). DNA damage induced by chronic inflammation contributes to colon carcinogenesis in mice. J. Clin. Invest..

[26] Wang LJ (2012). Interactome-wide analysis identifies end-binding protein 1 as a crucial component for the speck-like particle formation of activated absence in melanoma 2 (AIM2) inflammasomes. Mol. Cell. Proteomics.

[27] Wu YH (2014). Participation of c-FLIP in NLRP3 and AIM2 inflammasome activation. Cell Death Differ..

[28] Chen LC (2012). Tumour inflammasome-derived IL-1beta recruits neutrophils and improves local recurrence-free survival in EBV-induced nasopharyngeal carcinoma. EMBO Mol. Med..

[29] Cooper JA, Kaneko T, Li SS (2015). Cell regulation by phosphotyrosine-targeted ubiquitin ligases. Mol. Cell. Biol..

[30] Dou H (2012). Structural basis for autoinhibition and phosphorylation-dependent activation of c-Cbl. Nat. Struct. Mol. Biol..

[31] Buetow L (2016). Casitas B-lineage lymphoma linker helix mutations found in myeloproliferative neoplasms affect conformation. BMC Biol..

[32] Sanjay A (2001). Cbl associates with Pyk2 and Src to regulate Src kinase activity, alpha(v)beta(3) integrin-mediated signaling, cell adhesion, and osteoclast motility. J. Cell Biol..

[33] Haglund K, Ivankovic-Dikic I, Shimokawa N, Kruh GD, Dikic I (2004). Recruitment of Pyk2 and Cbl to lipid rafts mediates signals important for actin reorganization in growing neurites. J. Cell Sci..

[34] Zhou R, Yazdi AS, Menu P, Tschopp J (2011). A role for mitochondria in NLRP3 inflammasome activation. Nature.

[35] Salcedo R (2010). MyD88-mediated signaling prevents development of adenocarcinomas of the colon: role of interleukin 18. J. Exp. Med..

[36] Zaki MH, Vogel P, Body-Malapel M, Lamkanfi M, Kanneganti TD (2010). IL-18 production downstream of the Nlrp3 inflammasome confers protection against colorectal tumor formation. J. Immunol..

[37] Valimaki E, Miettinen JJ, Lietzen N, Matikainen S, Nyman TA (2013). Monosodium urate activates Src/Pyk2/PI3 kinase and cathepsin dependent unconventional protein secretion from human primary macrophages. Mol. Cell. Proteomics.

[38] Lietzen N (2011). Quantitative subcellular proteome and secretome profiling of influenza A virus-infected human primary macrophages. PLoS Pathog..

[39] Gao R (2015). Deletion of Src family kinase Lyn aggravates endotoxin-induced lung inflammation. Am. J. Physiol. Lung Cell. Mol. Physiol..

[40] Hafner-Bratkovic I, Pelegrin P (2018). Ion homeostasis and ion channels in NLRP3 inflammasome activation and regulation. Curr. Opin. Immunol..

[41] Frank GD, Motley ED, Inagami T, Eguchi S (2000). PYK2/CAKbeta represents a redox-sensitive tyrosine kinase in vascular smooth muscle cells. Biochem. Biophys. Res. Commun..

[42] Chapman NM, Houtman JC (2014). Functions of the FAK family kinases in T cells: beyond actin cytoskeletal rearrangement. Immunol. Res..

[43] Fan Y (2014). Cbl-b accelerates trypsin-induced cell detachment through ubiquitination and degradation of proline-rich tyrosine kinase 2. Tumour Biol..

[44] Wirnsberger G (2016). Inhibition of CBLB protects from lethal Candida albicans sepsis. Nat. Med..

[45] Youle RJ, van der Bliek AM (2012). Mitochondrial fission, fusion, and stress. Science.

[46] Sandhir R, Halder A, Sunkaria A (2017). Mitochondria as a centrally positioned hub in the innate immune response. Biochim. Biophys. Acta.

[47] Ramachandran R (2018). Mitochondrial dynamics: the dynamin superfamily and execution by collusion. Semin. Cell Dev. Biol..

[48] Lee JE, Westrate LM, Wu H, Page C, Voeltz GK (2016). Multiple dynamin family members collaborate to drive mitochondrial division. Nature.

[49] Kirisits A, Pils D, Krainer M (2007). Epidermal growth factor receptor degradation: an alternative view of oncogenic pathways. Int. J. Biochem. Cell Biol..

[50] Schroeder B, Weller SG, Chen J, Billadeau D, McNiven MA (2010). A Dyn2-CIN85 complex mediates degradative traffic of the EGFR by regulation of late endosomal budding. EMBO J..

[51] Koizumi Y (2012). Inflammasome activation via intracellular NLRs triggered by bacterial infection. Cell Microbiol..

[52] Shrivastava G, Leon-Juarez M, Garcia-Cordero J, Meza-Sanchez DE, Cedillo-Barron L (2016). Inflammasomes and its importance in viral infections. Immunol. Res..

[53] Menu P, Vince JE (2011). The NLRP3 inflammasome in health and disease: the good, the bad and the ugly. Clin. Exp. Immunol..

[54] Ghiringhelli F (2009). Activation of the NLRP3 inflammasome in dendritic cells induces IL-1beta-dependent adaptive immunity against tumors. Nat. Med..

[55] Kokubun H (2005). Determination of oxycodone and hydrocotarnine in cancer patient serum by high-performance liquid chromatography with electrochemical detection. Anal. Sci..

[56] Kawabata M, Kaneishi K (2013). Continuous subcutaneous infusion of compound oxycodone for the relief of dyspnea in patients with terminally ill cancer: a retrospective study. Am. J. Hosp. Palliat. Care.

[57] Ito K (2009). Effect of hydrocotarnine on cytochrome P450 and P-glycoprotein. Drug Metab. Pharmacokinet..

