# Supplementary material for: Src-family kinase-Cbl axis negatively regulates NLRP3 inflammasome activation
Source: Cell Death Dis. 2018 Oct 31;9(11):1109. doi: 10.1038/s41419-018-1163-z (PMC6208430; doi:10.1038/s41419-018-1163-z)
Supplement: Supplementary file 1 — Supplemental Material [file 41419_2018_1163_MOESM1_ESM.pdf]

Supplementary Information

Src-family kinase-Cbl axis negatively regulates NLRP3 inflammasome activation

I-Che Chung, Sheng-Ning Yuan, Chun-Nan OuYang, Hsin-Chung Lin, Kuo-Yang Huang, Yu-Jen Chen, An-Ko Chung, Ching-Liang Chu, David M. Ojcius, Yu-Sun Chang, Lih-Chyang Chen

Supplementary Contents

Supplementary Figure S1
Supplementary Figure S2
Supplementary Figure S3
Supplementary Figure S4
Supplementary Figure S5
Supplementary Figure S6

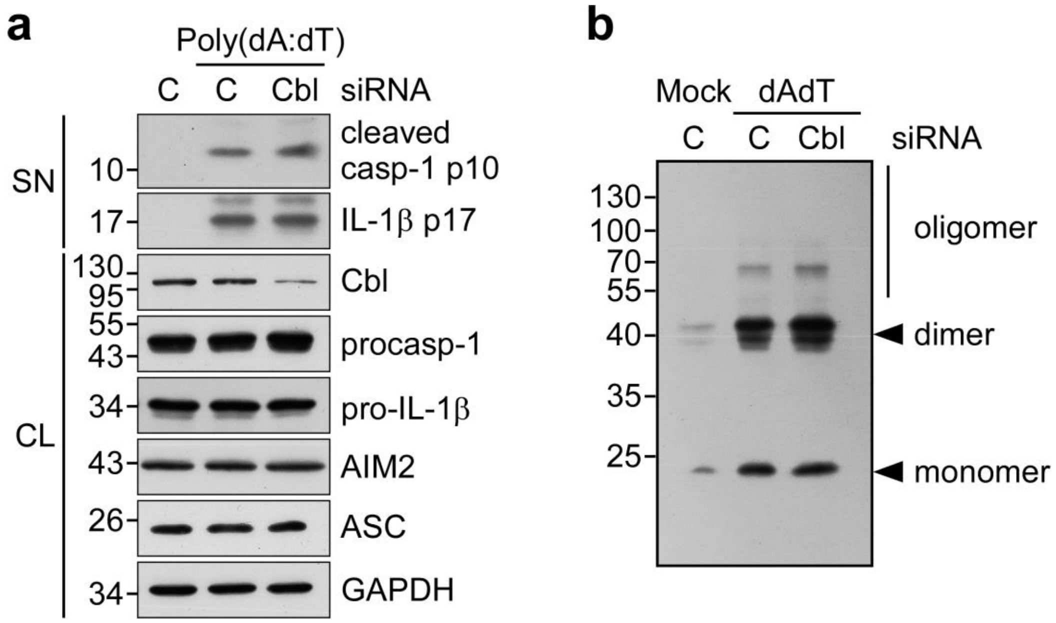

Supplementary Figure S1. Cbl does not regulate the AIM2 inflammasome. (a) THP-1-derived macrophages were transfected with Cbl siRNA or negative control (C) siRNA and treated with Poly(dA:dT) for 4 h, and culture supernatants (SN) and cell lysates (CL) were immunoblotted with antibodies that recognize Cbl, GAPDH and AIM2 inflammasome molecules. **(b)** Analysis of ASC oligomerization in THP-1-derived macrophages treated with Cbl siRNA or C siRNA, and then stimulated with Poly(dA:dT). The western blot is a representative of three independent experiments. Abbreviations: procasp-1, p45 precursor of caspase-1; cleaved Casp-1 p10, active caspase-1 subunits; IL-1β p17, secreted mature IL-1β; and pro-IL-1β, p31 precursor of IL-1β.

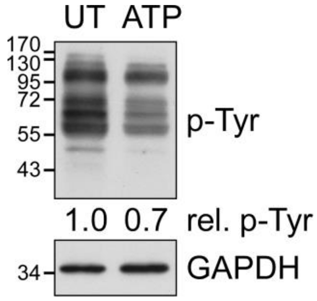

Supplementary Figure S2. ATP treatment decreases the global level of tyrosine-phosphorylated proteins. THP-1-derived macrophages were left untreated (UT) or treated with ATP for 4 h, and tyrosine-phosphorylated proteins (p-Tyr) were detected by immunoblotting with anti-p-Tyr.

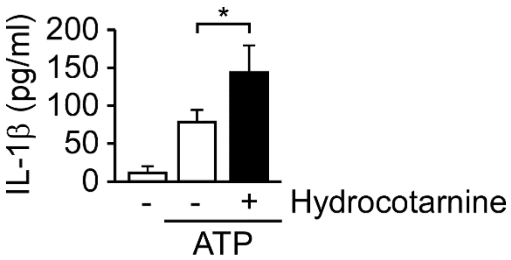

Supplementary Figure S3. Increase of IL-1β secretion by hydrocotarnine in THP-1 cells. THP-1-derived macrophages were pretreated with 10 μM hydrocotarnine for 1 h and then treated with ATP for 4 h, and supernatants were subjected to IL-1β and IL-18 ELISA. Induction of IL-18 secretion by ATP is failed to be detected. Symbols: *$P < 0.05$. All results are presented as the mean ± SD of three independent experiments, and were analyzed with the Student's t test.

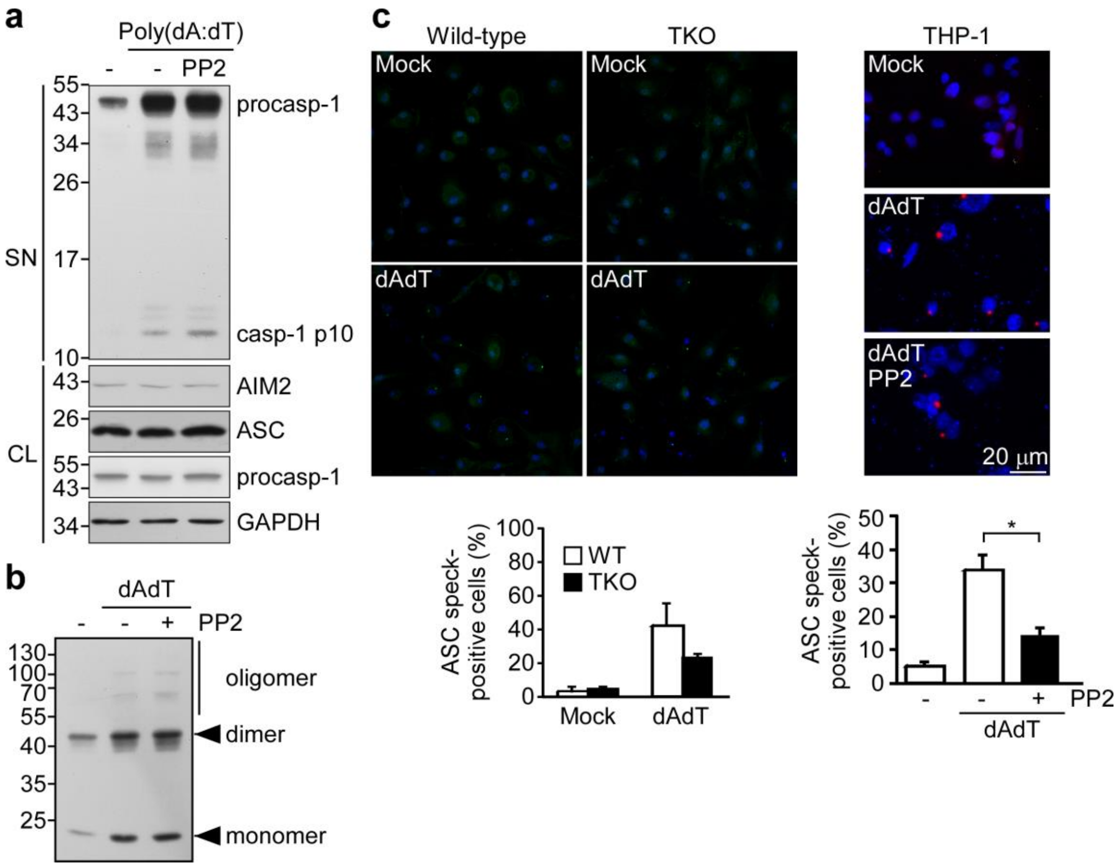

Supplementary Figure S4. SFKs do not regulate the AIM2 inflammasome. (a) THP-1-derived macrophages were pretreated with or without PP2 for 1 h and treated with Poly(dA:dT) for 4 h, and SN and CL were subjected with immunoblotting with antibodies that recognize GAPDH and AIM2 inflammasome molecules. **(b)** Analysis of ASC oligomerization in THP-1-derived macrophages that were pretreated with PP2 and then stimulated with Poly(dA:dT) for 4 h. The western blot is a representative of three independent experiments. **(c)** Left panel: BMDMs from WT and TKO mice were treated with Poly(dA:dT) for 4 h and ASC speck formation was visualized by immunostaining with an anti-ASC antibody. Right panel: PMA-differentiated ASC-mCherry-expressing THP-1 cells were pretreated with PP2 and then treated with Poly(dA:dT) for 4 h. ASC is shown in green (left panel) or red (right panel), while nuclei are blue. Scale bars, 20 μm. The percentage of cells with ASC specks, as determined using an IN Cell Analyzer (bottom panels). Abbreviations: procasp-1, p45 precursor of caspase-1; cleaved Casp-1, p10 active caspase-1 subunits. Symbol: $*P < 0.05$. All results are presented as the mean ± SD of three independent experiments, and were analyzed with the Student's t test.

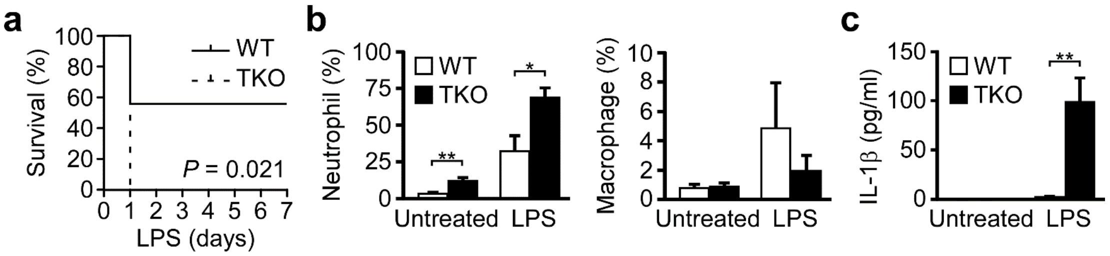

Supplementary Figure S5. SFKs protect mice from endotoxic shock. WT and TKO mice were challenged with intraperitoneal injection of LPS. **(a)** Survival rates in WT (n=9) and TKO (n=7) mice following intraperitoneal injection of 10 mg/kg LPS. **(b)** Percentage of $CD45^+$/$Ly6G^+$ neutrophils and $CD45^+$/$F4/80^+$ monocytes-macrophages in peripheral blood from WT (n = 5) and TKO (n = 5) mice at 24 h after 1 mg/kg LPS challenge. **(c)** ELISA of serum IL-1β in WT (n = 5) and TKO (n = 5) mice at 24 h after 1 mg/kg LPS challenge. Symbols: $*P < 0.05$; $**P < 0.01$.

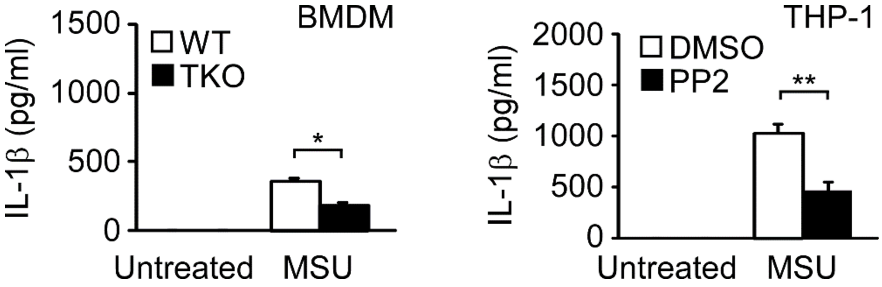

Supplementary Figure S6. SFKs are required for MSU-induced NLRP3 inflammasome activation. BMDMs from WT C57BL/6 and TKO mice (left panel) or PP2-preteated THP-1-derived macrophages (right panel) were treated with MSU for 4 h and supernatants were subjected to IL-1β ELISA. Symbols: *$P < 0.05$; **$P < 0.01$. All results are presented as the mean ± SD of three independent experiments, and were analyzed with the Student's t test.
